# The mini-PAT as a multi-source feedback tool for trainees in child and adolescent psychiatry: assessing whether it is fit for purpose

**DOI:** 10.1192/pb.bp.115.052720

**Published:** 2017-04

**Authors:** Gill Salmon, Lesley Pugsley

**Affiliations:** 1 Cwm Taf University Health Board, UK; 2 Cardiff University, UK

## Abstract

This paper discusses the research supporting the use of multi-source feedback (MSF) for doctors and describes the mini-Peer Assessment Tool (mini-PAT), the MSF instrument currently used to assess trainees in child and adolescent psychiatry. The relevance of issues raised in the literature about MSF tools in general is examined in relation to trainees in child and adolescent psychiatry as well as the appropriateness of the mini-PAT for this group. Suggestions for change including modifications to existing MSF tools or the development of a specialty-specific MSF instrument are offered.

Multi-source feedback (MSF) can motivate doctors to improve and change their practice.^1,2^ It gives doctors an overview of how others see them and compares this with their own view as well as the results of their peer group.^3^ MSF evolved in Canada and the USA out of a public demand for accountability to patients as well as an acceptance that assessments examining clinical decision-making and medical expertise do not address other essential competencies, such as interpersonal skills, professionalism and communication.^3^ MSF tools were originally designed to be formative, that is, to lead to awareness of and improvements in performance through feedback. More recently, however, they are being used for summative purposes, namely to provide information for revalidation and the annual review of competence progression (ARCP) which determines whether a trainee is considered fit to proceed with their training. As such, MSF tools need to be sufficiently reliable and valid. Reliability refers to the reproducibility of assessment measures or scores over repeated tests under identical conditions, and validity refers to the degree of confidence that an assessment measures what it is intended to measure. An associated term, feasibility, is a measure of whether an assessment instrument is practical, realistic and sensible given the circumstances and context.^4^

## Research on the use of MSF for doctors

Ramsey *et al*^5^ published a landmark study showing that it was feasible for internal medicine physicians to obtain peer assessments about their humanistic qualities, clinical practice and communication skills. They also came to important conclusions about the reliability of MSF – for example, that 11 peer ratings were needed to ensure a reliability coefficient of 0.7 (the minimum acceptable for workplace-based assessments (WPBAs)) and that the results were not substantially affected by the relationship between the rater and the person being rated nor by the method used to select the raters. The findings of this study also suggested that a doctor's medical knowledge (determined by examination marks) was not predictive of how peers subsequently rated their interpersonal relationships or communication skills.

The finding that reliable and valid MSF questionnaires can be developed and be feasible to use for assessing doctors has been replicated across settings and specialties.^2,6–9^ A number of systematic reviews have also been published, all of which conclude that MSF as a method of assessing communication skills, collegiality, humanism and professionalism in doctors has high reliability, validity and feasibility.^10–15^

## The mini-PAT as an MSF tool

The mini-PAT is used by the Royal College of Psychiatrists as an MSF instrument for trainees. It is well known because of its widespread use in the Foundation Programme.^16,17^ The mini-PAT was derived from the Sheffield Peer Review Assessment Tool (SPRAT) following a mapping exercise against the foundation curriculum,^6^ thus ensuring its content validity. The SPRAT contains 24 questions assessing a doctor's competencies and professional attributes, and it maps directly on to General Medical Council (GMC) standards of good medical practice,^18^ again establishing its content validity. These standards include good clinical care, maintaining good medical practice, teaching and training, appraising and assessing, relationships with patients and working with colleagues. The SPRAT was the first MSF tool validated in the UK for use by paediatric consultants as part of their appraisal.^19^ It has also been shown to be reliable, needing as few as four raters to determine whether a doctor is in difficulty or not (more in borderline situations), and feasible, taking only 5–6 minutes to use with good return rates (more than 70%).^20^ It can also discriminate between the more and less experienced trainee.^21^

In developing the mini-PAT, nine questions which did not map on to the curriculum for the Foundation Programme were removed from the SPRAT. These included questions relating to the management of complex patients and leadership. One question about probity and health was added while the free-text element and six-point scale (where 1 indicates ‘very poor’ and 6 indicates ‘very good’) remained unchanged.^6^ The resulting mini-PAT was thought to reflect the importance for foundation doctors of developing communication skills, team work and other humanistic qualities in relation to patient care in addition to their medical knowledge.^3^

In his critical analysis of the mini-PAT, while accepting its content validity and feasibility, Abdulla stated that it ‘lacks sufficient field evaluation and has not gone through any stringent criteria that are required for the validation of an assessment tool’.^3^ Data on the reliability and validity of 693 mini-PAT assessments on 553 foundation year 1 and 2 (F1/F2) doctors have subsequently been published.^6^ The mean scores of the two groups were found to be significantly different when using the same criterion standard (i.e. expectation for F2 completion), with 19.6% of F1s and 5.6% of F2s being assessed as borderline or below the expectations for F2 completion. This was used as evidence of internal standardisation and construct validity, as was the finding that the trainees scored higher in the domains of working with colleagues and relationships with patients compared with the clinical skills domains. Overall, 53% of F1 doctors and 74% of F2 doctors could have been assessed by no more than 8 assessors based on their mean scores. Factor analysis revealed that the two main factors were humanistic qualities and clinical performance. The authors concluded that the mini-PAT was a valid and reliable MSF tool for assessing foundation doctors.

## Use of the mini-PAT in child and adolescent psychiatry training

In child and adolescent psychiatry, the process when using the mini-PAT is as follows: twice a year, the trainee provides contact details of between 8 and 12 co-workers who see them on a frequent basis in a range of situations. These people and the trainee then complete the mini-PAT online. Presumably based on the findings of Archer *et al*,^6^ it is suggested that at least 8 forms must be completed to ensure the assessment is reliable. There is, however, no research specifically related to the mini-PAT on the minimum number of assessors required to give a valid result.^3^ The form uses a 6-point Likert-type rating scale. Trainees are rated according to the standard expected at each stage of training. A score of 4 corresponds to the expected standard, with higher or lower scores suggesting the trainee's performance is better or worse.^22^ The responses are analysed centrally and a report is then sent to the trainee's educational supervisor who delivers the feedback in person.^23^

## Potential issues with using MSF tools

Several issues that have been identified in relation to the use of MSF tools for medical practitioners in general are also relevant to their use in child and adolescent psychiatry. One is the trainee's choice of rater. Although several authors have found that MSF assessment is not necessarily biased by allowing the doctor to select their own raters,^5,24,25^ others have found that factors such as the seniority, gender and profession of raters can significantly influence the assessment. For example, Archer *et al*^21^ found that consultant raters using the SPRAT gave significantly lower mean scores to paediatric trainees than more junior doctors did; similarly, Bullock *et al*^26^ found that consultants and senior nurses were more likely to give ‘concern’ ratings when assessing junior doctors than were peers or administrators. Thus, there is a trend for assessors to be more critical with increasing seniority. When considering the mini-PAT, Archer *et al*^6^ found that assessors' scores were affected by their occupation, the length of time the trainee had been working with them, and the working environment. They suggested standardising the number of consultants used as raters by each trainee. These findings support the need for more detailed guidance in rater selection from the Royal College of Psychiatrists. Trainees are currently only advised that raters be chosen from a broad range of co-workers.^4^ In addition, Abdulla^3^ suggests that selection bias can be reduced if the list of raters is discussed and agreed on beforehand with the trainee's supervisor.

Measurement errors, such as the central tendency and halo effect, can also occur and are particularly likely when behaviours which cannot be easily observed are being assessed.^27^ A particular issue for non-doctor raters is knowing what standards they should expect for a doctor at that stage in their training. In an attempt to reduce measurement errors, Abdulla^3^ suggests better education for mini-PAT raters. This could be provided by the Royal College of Psychiatrists as part of their online mini-PAT package.

It has been shown that doctors' self-assessments do not correlate well with peer or patient ratings.^7,28^ Violato & Lockyer^29^ studied psychiatrists, internal medicine physicians and paediatricians, and found that all were inaccurate in assessing their own performance. Those psychiatrists who were rated by peers to be in the bottom quartile saw themselves as ‘average’, whereas the psychiatrists in the top quartile significantly underrated themselves. This indicates that poorly performing doctors often lack insight, not always accepting negative feedback from others and querying its validity.^30^ Overeem *et al*^31^ advise that trained facilitators should encourage trainees to reflect on MSF results and help them set concrete goals for improvement. Offering coaching to help trainees identify their strengths and weaknesses may help facilitate changes in performance.^32^ Making the feedback highly structured can help trainees acknowledge feedback from all sources rather than just the medical scores which they tend to value more.^5,33–35^ Although taking the mean of the scores may be the most reliable approach,^36^ attention should also be given to the free-text comments which might highlight specific performance issues and which may also make the feedback more acceptable.^35^ These findings highlight the importance of the MSF feedback process, which should include the development of a relevant action plan in collaboration with the doctor.

It has been proposed that a single, generic MSF tool be used in the UK.^37^ Research supporting this includes Violato & Lockyer's^29,38^ study of the use of one MSF tool for internal medicine physicians, paediatricians and psychiatrists. Although they found no specialty differences in response rates or reliability, it is of note that of the items clustered into the same four factors across the specialties, for psychiatry the most discriminating factor was communication whereas for the other two specialties the most important was patient management. By contrast, Mackillop *et al*^39^ evaluated the use of a generic MSF tool across specialties and concluded that, although the generic content was appropriate for most specialties, some would benefit from specialty-specific content.

## Does the mini-PAT suit the needs of trainees in child and adolescent psychiatry?

In child and adolescent psychiatry, the mini-PAT is currently used to assess trainees. Although the mini-PAT has content validity for foundation doctors, having been mapped against their curriculum, this does not necessarily mean it is also a valid tool for other grades or for use across specialties. In the making of the mini-PAT, some questions were removed from the SPRAT, namely those relating to management of complex patients and leadership.^6^ However, these items are highly relevant to trainees in child and adolescent psychiatry. Davies *et al*^40^ modified the SPRAT for trainees in histopathology following a blueprinting exercise against the histopathology curriculum to establish content validity. They concluded that specialty-specific MSF is feasible and achieves satisfactory reliability. A similar approach blueprinting the SPRAT against the child and adolescent psychiatry competency-based curriculum^41^ could therefore be considered. The SPRAT also requires fewer raters than the mini-PAT in order for the results to be sufficiently reliable,^6^ thus adding to its potential suitability for child psychiatry trainees who often work in small teams.

Alternatively, a specialty-specific MSF instrument for child and adolescent psychiatry trainees could be developed, to reflect the differences in their practice compared with other specialties and the greater importance placed on communication, interpersonal skills, emotional intelligence and relationship building.^4^ Tools taking these attributes into account have been developed for use with consultant psychiatrists and have been found to be feasible to use as well as being reliable and valid.^42,43^ The child and adolescent psychiatry competency-based curriculum^41^ gives details of intended learning outcomes (ILOs), which are either mandatory or selective, some of which tap into these areas. The ILOs range from those that are predominantly clinical (e.g. managing emergencies (mandatory), paediatric psychopharmacology (mandatory) and paediatric liaison (selective)) to those that focus on more humanistic skills (e.g. professionalism (mandatory) and establishing and maintaining therapeutic relationships with children, adolescents and families (mandatory)). The ILO on professionalism includes: ‘practicing Child and Adolescent Psychiatry in a professional and ethical manner; child and family centred practice; understanding the impact of stigma and other barriers to accessing mental health services and inter-professional and multi-agency working’.^41^ Some of the necessary associated skills which trainees are expected to attain include: supervising junior psychiatric staff, working with colleagues within the team and with other agencies to put the child's needs as central, and acting as an advocate for the child. There is scope to develop this area of the curriculum even further; the American Board of Pediatrics (ABP) published guidelines for the teaching and evaluation of professionalism in paediatric residency programmes^44^ as well as standards of professional behaviour against which paediatricians, including those in training, can be evaluated.^45^ Both are of relevance to child and adolescent psychiatrists.

If developed, a child and adolescent psychiatry specialty-specific MSF instrument would need to map on to the relevant ILOs. It could also include feedback from patients and families (which is not currently routinely collected as part of the WPBAs) to reflect the need to balance the views of the child (who is the patient) with those of their carers.

## Conclusions

MSF tools such as the mini-PAT can provide reliable and valid information on areas of a trainee's performance such as communication skills and other humanistic qualities affecting patient care for which other forms of assessment, such as written examinations, are unhelpful. MSF tools have their predominant strength when used for formative assessment and were generally designed for this purpose. They are most appropriately used within a portfolio of other WPBAs and can help in making decisions about a doctor's fitness to practice or to continue training.^46^ Rater bias and measurement error could be reduced by offering more detailed guidance to trainees in their choice of rater as well as to raters in the use of the tool. Measurement error could also be reduced by encouraging trainees to obtain a larger number of returns than the minimum of eight recommended by the Royal College of Psychiatrists.^3^ The quality of the feedback to the trainee is also important and educational supervisors would benefit from training in this area.

Although the mini-PAT is used widely across specialties, it has only been properly evaluated for use with foundation doctors. Interested researchers, clinicians or educationalists might now want to consider developing a modified version of the SPRAT or a specialty-specific MSF tool that is more appropriate for the needs of trainees in child and adolescent psychiatry. This would reflect the differences in their day-to-day practice compared with that of other trainees but would obviously need to be mapped to the curriculum and evaluated in practice to ensure content validity and reliability.
